# Hyperreflective Foci and Subretinal Fluid Are Potential Imaging Biomarkers to Evaluate Anti-VEGF Effect in Diabetic Macular Edema

**DOI:** 10.3389/fphys.2021.791442

**Published:** 2021-12-23

**Authors:** Shiyue Qin, Chaoyang Zhang, Haifeng Qin, Hai Xie, Dawei Luo, Qinghua Qiu, Kun Liu, Jingting Zhang, Guoxu Xu, Jingfa Zhang

**Affiliations:** ^1^Department of Ophthalmology, the Second Affiliated Hospital of Soochow University, Suzhou, China; ^2^Department of Ophthalmology, Taizhou People’s Hospital, Taizhou, China; ^3^Department of Ophthalmology, Shanghai General Hospital (Shanghai First People’s Hospital), Shanghai Jiao Tong University, Shanghai, China; ^4^National Clinical Research Center for Eye Diseases, Shanghai Key Laboratory of Ocular Fundus Diseases, Shanghai Engineering Center for Visual Science and Photomedicine, Shanghai Engineering Center for Precise Diagnosis and Treatment of Eye Diseases, Shanghai, China; ^5^Department of Ophthalmology, Changhai Hospital, Shanghai, China; ^6^Department of Regenerative Medicine, Tongji Eye Institute, Tongji University School of Medicine, Shanghai, China; ^7^Department of Pharmacology, Tongji Eye Institute, Tongji University School of Medicine, Shanghai, China; ^8^Department of Ophthalmology, Shigatse People’s Hospital, Xizang, China

**Keywords:** diabetic macular edema, anti-VEGF, OCT angiography, inflammation, hyperreflective foci, cystoid edema, subretinal fluid

## Abstract

**Purpose:** The aim was to investigate the effect and underlying mechanism of anti-vascular endothelial growth factor (anti-VEGF) in diabetic macular edema (DME) by optical coherence tomography angiography (OCTA).

**Methods:** Twenty-five eyes in 18 treatment-naïve patients with DME were included. All eyes were imaged by OCTA at baseline and 1 week after monthly intravitreal aflibercept injection (IAI). Visual acuity was measured as best corrected visual acuity (BCVA). Additional parameters were evaluated by OCTA, including central macular thickness (CMT), the number of hyperreflective foci (HRF), foveal avascular zone (FAZ), vessel density (VD) in the deep capillary plexus (DCP), the en-face area of cystoid edema in DCP segmentation, and subretinal fluid (SRF) height.

**Results:** The mean time between baseline and final follow-up by OCTA was 79.24 ± 38.15 (range, 28–163) days. Compared with baseline, BCVA was increased significantly after the 3rd IAI, while CMT was decreased significantly from the 1st IAI. SRF height and the area of cystoid edema in DCP segmentation were decreased significantly after the 2nd IAI compared with baseline. The number of HRF was decreased significantly after the 1st IAI (8.87 ± 9.38) compared with baseline (11.22 ± 10.63). However, FAZ’s area and perimeter as well as VD in DCP showed no significant changes post-treatment.

**Conclusion:** Anti-VEGF is effective in treating DME, improving visual acuity and decreasing macular edema. The decreased HRF indicates anti-inflammatory effects of aflibercept to deactivate retinal microglia/macrophages. The decreased cystoid edema and SRF height indicated improved drainage function of Müller glial cells and retinal pigment epithelium after IAI.

## Highlights

1. Diabetic macular edema is a major cause of visual loss in patients with diabetic retinopathy, seriously affecting the patient’s quality of life.
2. Currently, anti-VEGF treatment has become the standard of care and first-line therapy for the treatment of diabetic macular edema.
3. The inflammation and drainage dysfunction accounts for the pathogenesis of diabetic macular edema.
4. Aflibercept, *via* binding vascular endothelial growth factor (VEGF) and placental growth factor (PlGF), effectively decreased macular edema by deactivating microglia and improving the drainage function of retinal Müller glia and retinal pigment epithelium.

## Introduction

Diabetic macular edema (DME), a major cause of central visual loss in patients with diabetic retinopathy, has a prevalence of 4.8% and seriously affects the patient’s quality of life (Varma et al., 2014). The pathological basis of DME encompasses the breakdown of the blood–retinal barrier (BRB) and concomitant neuroglial dysfunction (Yoshitake et al., 2019). Disruption of the BRB, including inner and outer BRB, induces an influx of fluid into the retina, resulting in intraretinal and subretinal fluid accumulation (Bhagat et al., 2009). Meanwhile, the dysfunction of retinal Müller glia (RMG) and retinal pigment epithelium (RPE) results in cystoid edema and subretinal fluid accumulation (Daruich et al., 2018). Although the exact pathophysiological mechanism of DME remains unclear, it is currently assumed that macular edema results from an imbalance between fluid entry and fluid exit driven by the Starling equation (Daruich et al., 2018).

In recent years, intravitreal injections of anti-vascular endothelial growth factor (anti-VEGF) drugs have revolutionized the treatment of DME, becoming the standard of care and first-line therapy (Wells et al., 2015). Currently, anti-VEGF drugs, including bevacizumab (Avastin), ranibizumab (Lucentis), aflibercept (Eylea), and conbercept (Lumitin), are potent in the treatment of DME. Among these, bevacizumab and ranibizumab bind VEGF-A, while aflibercept and conbercept bind VEGF-A, VEGF-B, and placental growth factor (PlGF). Although most DME patients significantly benefit from anti-VEGF treatment, about 31–66% still show poor response to monthly anti-VEGF treatment (Bressler et al., 2018), indicating that beyond VEGF, other pathogenic factors are also involved in the pathogenesis of DME, including microglial activation, and RMG and RPE dysfunction.

Recently, hyperreflective foci (HRF) in the retina have been considered active inflammatory cells, especially microglia and macrophages, when examined with optical coherence tomography (OCT), indicating inflammation also plays an important role in the pathogenesis of retinal diseases. HRF were first described by Coscas et al. (2013) as hyperreflective dots (HRD) by spectral-domain OCT in patients with age-related macular degeneration. Subsequently, HRF have been reported in many retinal diseases, including diabetic retinopathy and DME, retinal vein occlusion, choroideremia, and other retinal degenerative diseases (Vujosevic et al., 2017; Frizziero et al., 2020; Romano et al., 2020). Although there is still no consensus on their origin, HRF likely reflect a progressive state of damaged retinal tissue within an inflammatory retinal microenvironment.

Both RMG and RPE are important in clearing both intraretinal and subretinal fluids from the retina *via* the corresponding ion and water channels, thus maintaining the retina in a relatively dehydrated, transparent state. The dysfunction of RMG and RPE results in fluid retention in the retina, i.e., intraretinal cystoid edema due to RMG drainage dysfunction, and subretinal fluid (SRF) accumulation due to RPE dysfunction (Daruich et al., 2018). Optical coherence tomography angiography (OCTA) is a noninvasive, depth-resolved imaging technique, which allows the visualization of retinal microstructure in both B-scan and en-face modes, as well as the quantification of vascular density (VD), foveal avascular zone (FAZ), and non-perfusion area, etc., in the designated area. In our clinical practice, decreased HRF, cystoid edema and SRF were observed in patients with DME after anti-VEGF treatment, indicating that besides maintaining the integrity of the BRB, anti-VEGF drugs might exert additional effects on other retinal cells, such as microglia and/or macrophages, RMG and RPE.

In this study, we retrospectively investigated 25 eyes in 18 treatment-naïve patients with DME before and after treatment with special focus on the changes of HRF, cystoid edema, and SRF as assessed by OCTA. The data showed that aflibercept is effective in treating DME, resulting in increased visual acuity and decreased CMT. The decreased number of HRF after intravitreal aflibercept injection (IAI) treatment indicated that aflibercept might deactivate microglia/macrophages. The decreased cystoid edema indicated aflibercept improves the drainage function of RMG; the reduced SRF suggested that aflibercept improves the drainage function of RPE and maintains the barrier integrity of the BRB. These data jointly indicated that besides maintaining BRB to reduce DME, aflibercept treatment could also decrease retinal inflammation and improve drainage function in both RMG and RPE *via* binding VEGF and PlGF.

## Patients and Methods

### Patients

Twenty-five treatment-naïve eyes in 18 patients with DME were diagnosed by comprehensive ophthalmologic examinations in the Department of Ophthalmology, Shanghai General Hospital affiliated to Shanghai Jiao Tong University, Shanghai, China, between July 2020 and December 2020. This study was approved by the Clinical Research Ethical Committee of Shanghai General Hospital affiliated to Shanghai Jiao Tong University (Permit No. 2020KY205) and adhered to the principles of the Declaration of Helsinki. All individual participants provided written informed consent.

All patients underwent routine ophthalmological examinations before and after treatment, including best corrected visual acuity (BCVA), intraocular pressure (IOP), slit-lamp biomicroscope, and fundus examinations. Visual acuity was presented as the logarithm of the minimum angle of resolution (logMAR). Re-evaluation was performed 1 week after each IAI.

Inclusion criteria were: >18 years of age; central macular thickness (CMT) >300 μm; intravitreal injections administered monthly until OCTA showing no significant macular edema; and OCTA performed at baseline and 1 week after each IAI, with complete and high-quality images.

Exclusion criteria were: other retinal diseases, including severe proliferative diabetic retinopathy, retinal vein occlusion, neovascular age-related macular edema, and high myopia; recent history of intraocular surgery for cataract, glaucoma or eye trauma, vitreous hemorrhage, or tractional or rhegmatogenous retinal detachment.

### Intravitreal Aflibercept Injection

An experienced ophthalmologist performed all intravitreal injections, at the temporal limbus through the eyeball’s pars plana under aseptic conditions. All patients received monthly intravitreal injections of aflibercept (2 mg/0.05 ml) using a 30-gauge needle. A 1-week variation was allowed for every injection interval.

### Optical Coherence Tomography Angiography

The retina was imaged on the RTVue XR Avanti OCT system (Optovue, Inc., Fremont, CA, United States), and the parameters of interest were acquired with the AngioVue software. The scan covered an area of 6 mm × 6 mm centered on the fovea. Enface images of the superficial capillary plexus (SCP), deep capillary plexus (DCP), FAZ, vessel density (VD), etc., were automatically recorded and analyzed by the OCTA auto-segmentation software. The analysis was performed as previously described by our team (Wu et al., 2021).

**Figure 1:**
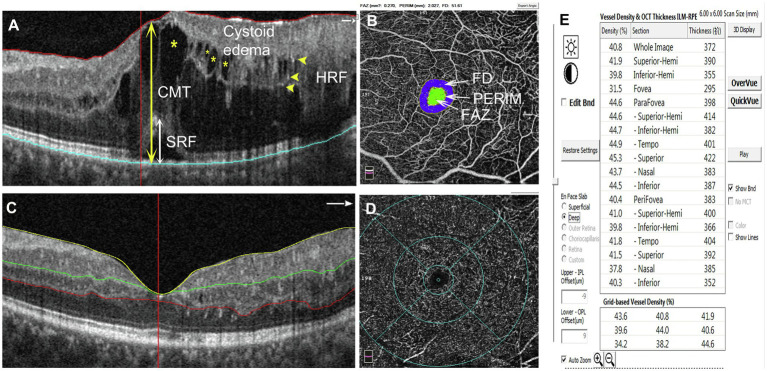
Schematic diagram showing the detected parameters. **(A)** The measurements of CMT and the height of SRF are indicated with double-head arrows (CMT: yellow, SRF: white). HRF are shown by yellow arrowheads. Cystoid edemas are marked with yellow asterisks. **(B)** The inner circle (green area) indicates the FAZ area; PERIM is the circumference of the FAZ; FD indicates foveal blood flow density within the blue area. The parameters can be read directly on the image. **(C)** SCP segmentation was between the ILM (yellow line) and 15 μm below the IPL (green line). DCP segmentation was between 15 μm below the IPL (green line) and 70 μm below the IPL (red line). **(D, E)** Selecting the density option for the VD parameters of different retinal partitions. CMT, central macular thickness; SRF, subretinal fluid; HRF, hyperreflective foci; FAZ, foveal avascular zone; FD, foveal blood flow density; SCP, superficial capillary plexus; ILM, internal limiting membrane; IPL, inner plexiform layer; and DCP, deep capillary plexus.

The following parameters were evaluated: CMT, the number of HRF, en-face area of cystoid edema in DCP segmentation, the height of SRF, FAZ, and VD in DCP (Figure 1). CMT, FAZ, and VD were analyzed automatically with the software on OCTA. HRF were counted manually in the entire retina within a length of 6 mm. The height of SRF in the subfovea was manually measured with the ImageJ software. The en-face area of cystoid edema in DCP segmentation was measured with the ImageJ software by a method aimed at isolating the contour of cysts from the surrounding tissue. First, the image was converted into the eight-bit mode and then binarized with a threshold of 30% of maximum brightness. The threshold of 30% was chosen after evaluating and comparing cysts’ darkness in different images and defining the threshold for discriminating between cysts and the surrounding tissue. Finally, the area of the selected region was measured and indicated as pixels.

### Statistical Analysis

The SPSS 23.0 software was applied for statistical analysis. Data are mean ± SD. Independent samples *t* test was performed for between-group comparisons. Correlations were assessed by Pearson correlation analysis. Value of *p* < 0.05 was considered statistically significant.

## Results

### Baseline Characteristics of the Patients

Eighteen (18) treatment-naïve patients with DME, including eight females (44%) and 10 males (56%), were enrolled in this retrospective cohort study, in which 25 eyes were analyzed, i.e., 10 eyes from females (40%) and 15 eyes from males (60%). The patients were 63.2 ± 12.55 years old (range, 30–83 years). One eye in 11 patients and two eyes in seven patients were enrolled. DME was classified as cystoid macular edema (17 eyes) and subretinal fluid (eight eyes). Totally 25 (1st IAI), 19 (2nd IAI), seven (3rd IAI), and four (4th IAI) eyes completed the follow-ups, respectively, 1 week after each intravitreal injection (Table 1).

**Table 1:** Baseline characteristics of the patients.

Number of patients	18
Number of studied eyes	25
Patients with single eyes examined	11
Patients with both eyes assessed	7
**Gender**	
Female (%)	8 (44.44)
Male (%)	10 (55.56)
**Mean age (years)**	63.20 ± 12.55
**Type of DME**	
Cystoid edema (%)	17 (68.0)
SRF (%)	8 (32.0)
**Follow-up**	
Baseline	25
1st IAI	25
2nd IAI	19
3rd IAI	7
4th IAI	4

Data are number or mean ± SD. DME, diabetic macular edema; SRF, subretinal fluid; and IAI, intravitreal aflibercept injection.

### Best Corrected Visual Acuity Is Improved Significantly After IAI

**Figure 2:**
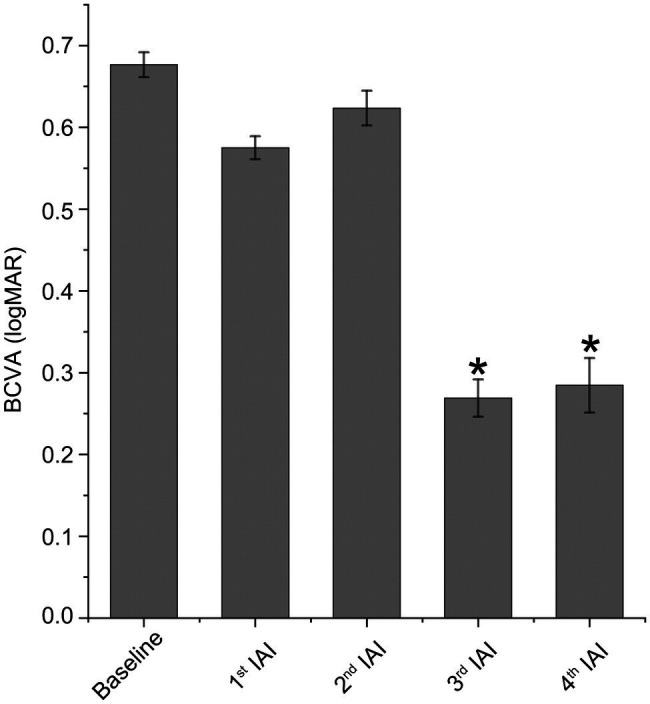
Visual acuity is improved after IAI. BCVA was expressed as logMAR. Data are mean ± SD. *n* = 21 (baseline), 21 (1st IAI), 17 (2nd IAI), 4 (3rd IAI) and 3 (4th IAI). ^*^p < 0.05 vs. baseline. BCVA, best corrected visual acuity and IAI, intravitreal aflibercept injection.

Table 2 summarizes logMAR BCVA differences between study variables at baseline and 1 week after monthly injections. Mean BCVA was improved after each IAI. As shown in Table 2 and Figure 2, compared with baseline (logMAR 0.63 ± 0.28), visual acuity was gradually improved after the 1st and 2nd injections and improved significantly after the 3rd (logMAR 0.27 ± 0.10, *p* < 0.05) and 4th (logMAR 0.28 ± 0.12, *p* < 0.05) injections.

**Table 2:** BCVA values before and after IAI in patients with DME.

Follow-up	Mean ± SD (logMAR)	*n*	95% CI	*p*
Baseline	0.63 ± 0.28	21	-	-
1st IAI	0.58 ± 0.30	21	0.02, 0.13	0.171
2nd IAI	0.62 ± 0.37	17	−0.09, 0.14	0.668
3rd IAI	0.27 ± 0.10	4	0.06, 0.72	0.033
4th IAI	0.28 ± 0.12	3	0.12, 0.51	0.020

Data are number or mean ± SD. Statistically analysis was performed between baseline and each IAI. *p* < 0.05 was considered statistically significant. BCVA, best corrected visual acuity; LogMAR, the logarithm of the minimum angle of resolution; IAI, intravitreal aflibercept injection; and CI, confidence interval.

### Central Macular Thickness Is Decreased Significantly After IAI

**Figure 3:**
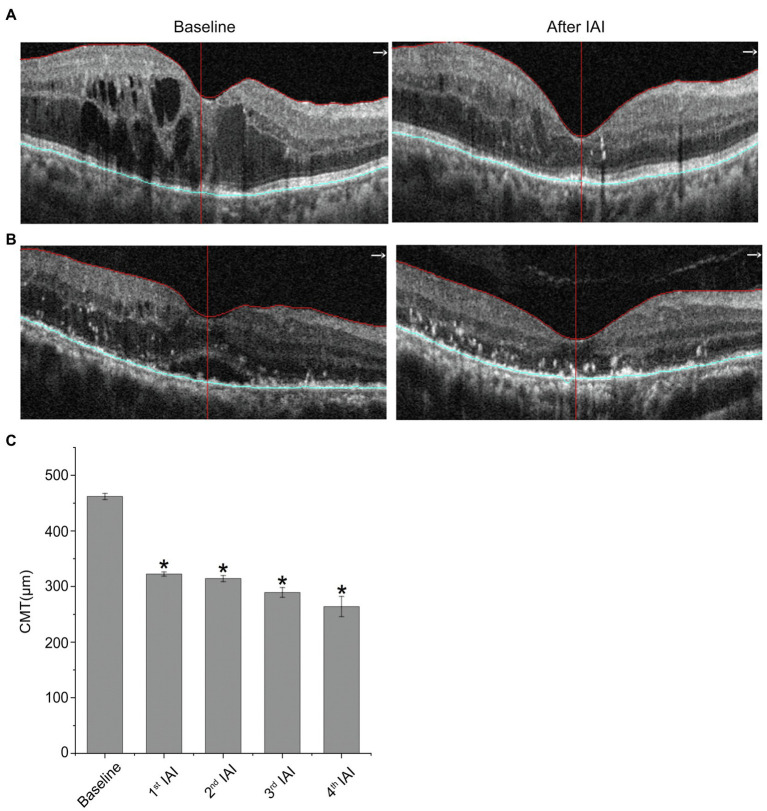
CMT is decreased after IAI. **(A)** Representative images of a 77-year-old female patient with DME at baseline and after a monthly IAI. **(B)** Representative images of a 70-year-old male patient with DME at baseline and after a monthly IAI. **(C)** Decreased CMT following IAI. Data are mean ± SD. ^*^p < 0.05 vs. baseline. CMT, central macular thickness and IAI, intravitreal aflibercept injection.

Table 3 and Figure 3 reveal decreased CMT after IAI. Compared with baseline (462.00 ± 129.67 μm), CMT was decreased significantly (*p* < 0.05) after the 1st (328.48 ± 91.00 μm), 2nd (321.41 ± 98.08 μm), 3rd (289.43 ± 66.03 μm), and 4th (264.00 ± 83.92 μm) injections. Statistical analysis between 1st IAI and subsequent IAIs was also performed. The results showed that, compared with 1st IAI (328.48 ± 91.00 μm), CMT was decreased after the 2nd (321.41 ± 98.08 μm, *p* = 0.949), 3rd (289.43 ± 66.03 μm, *p* = 0.250), and 4th (264.00 ± 83.92 μm, *p* = 0.057) injections. Although there was a trend for CMT decrease after the following IAIs, no statistical difference for CMT change was found between 1st IAI and subsequent IAIs, indicating no additive effect of IAI on CMT improvement possibly due to the small sample size and variations for CMT change in each patient.

**Table 3:** CMT values before and after IAI in patients with DME.

Follow-up	Mean ± SD (μm)	*n*	95% CI	*p*
Baseline	462.00 ± 129.67	23	-	-
1st IAI	328.48 ± 91.00	23	64.43, 202.61	0.001
2nd IAI	321.41 ± 98.08	17	38.63, 169.85	0.004
3rd IAI	289.43 ± 66.03	7	44.30, 287.70	0.016
4th IAI	264.00 ± 83.92	4	27.58, 312.42	0.032

Data are number or mean ± SD. Statistically analysis was performed between baseline and each IAI. *p* < 0.05 was considered statistically significant. CMT, central macular thickness; IAI, intravitreal aflibercept injection; and CI, confidence interval.

### The Height of Subretinal Fluid Is Decreased Significantly After IAI

**Figure 4:**
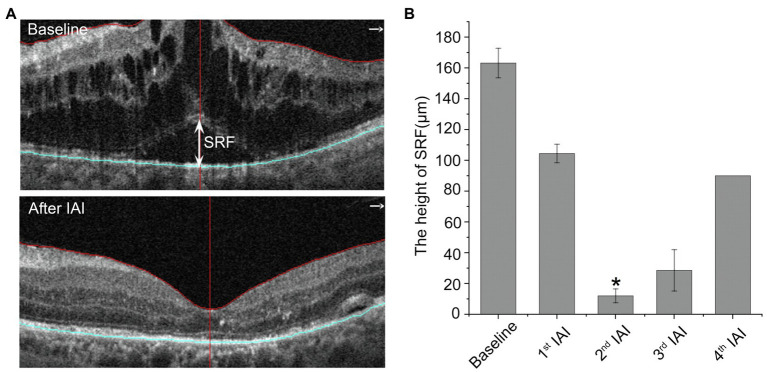
The height of SRF is decreased after IAI. **(A)** Representative images of a 50-year-old male DME patient with SRF at baseline and after a monthly IAI. The double-head arrow indicates the SRF. **(B)** Decreased height of SRF following IAI. Data are mean ± SD. ^*^p < 0.05 vs. baseline. SRF, subretinal fluid and IAI, intravitreal aflibercept injection.

SRF reflects RPE dysfunction, including outer BRB breakdown and decreased drainage function. To examine whether aflibercept could enhance the clearance of SRF, the heights of SRF were measured before and after IAI. As shown in Table 4 and Figure 4, compared with baseline (163.16 ± 82.09 μm, *n* = 8), the heights of SRF were decreased gradually after IAI, i.e., 104.43 ± 51.17 μm (1st IAI, *n* = 8, *p* = 0.150), 11.98 ± 29.36 μm (2nd IAI, *n* = 6, *p* = 0.011), 28.53 ± 49.42 μm (3rd IAI, *n* = 3, *p* = 0.267), and 45.00 ± 63.64 μm (4th IAI, *n* = 2, *p* = 0.500), respectively. Surprisingly, the heights of SRF were increased after the 3rd and 4th injections compared with the 2nd injection, possibly due to the small sample size (*n* = 2–3) and the recurrence of SRF in these patients.

**Table 4:** SRF before and after IAI in patients with DME.

Follow-up	Mean ± SD (μm)	*n*	95% CI	*p*
Baseline	163.16 ± 82.09	8	-	-
1st IAI	104.43 ± 51.17	8	−27.23, 144.67	0.15
2nd IAI	11.98 ± 29.36	6	42.48, 199.53	0.011
3rd IAI	28.53 ± 49.42	3	−129.89, 272.34	0.267
4th IAI	45.00 ± 63.64	2	−292.66, 342.66	0.5

Data are number or mean ± SD. Statistically analysis was performed between baseline and each IAI. *p* < 0.05 was considered statistically significant. SRF, subretinal fluid; IAI, intravitreal aflibercept injection; and CI, confidence interval.

### Retinal Cystoid Edema Is Decreased Significantly After IAI

**Figure 5:**
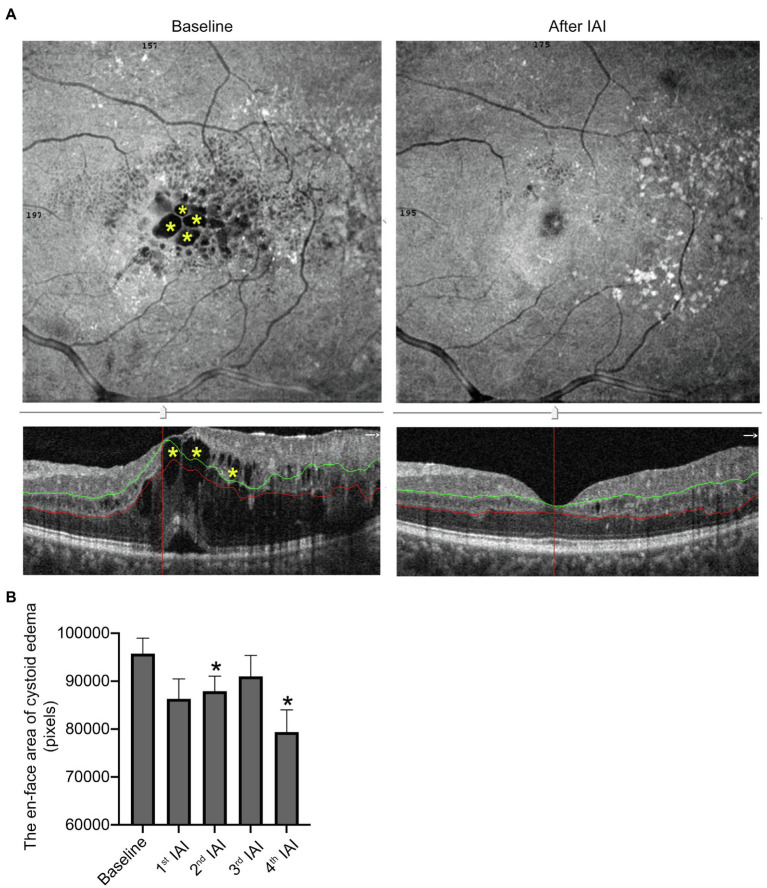
The area of cysts in DCP is decreased after IAI. **(A)** Representative en-face and B-scan (yellow asterisk) OCTA images of an 84-year-old male patient with cystoid edema in DCP at baseline and after a monthly IAI. **(B)** Decreased en-face area of cysts in DCP following IAI. Data are mean ± SD. ^*^p < 0.05 vs. baseline. DCP, deep capillary plexus; OCTA, optical coherence tomography angiography; and IAI, intravitreal aflibercept injection.

Retinal cystoid edema indicates the drainage dysfunction of RMG due to the misdistribution and decreased expression levels of ion and water channels, including inwardly rectifying K^+^ channel 4.1 (Kir4.1) and aquaporin 4 (AQP4), affected by diabetes (Daruich et al., 2018). To examine the change of cystoid edema after IAI, the en-face areas of cystoid edema were assessed before and after each IAI by OCTA on the auto-segmentation of DCP. The data showed that compared with baseline (95760.94 ± 12913.65 pixels), the en-face area of cystoid edema on DCP segmentation was decreased gradually after IAI, i.e., 86307.06 ± 16719.92 pixels (1st IAI, *n* = 16, *p* = 0.100), 87927.18 ± 10398.46 pixels (2nd IAI, *n* = 11, *p* = 0.003), 91020.40 ± 13764.53 pixels (3rd IAI, *n* = 10, *p* = 0.059), and 79397.17 ± 11293.08 pixels (4th IAI, *n* = 6, *p* = 0.007; Figure 5; Table 5). The decreased cystoid edema after IAI indicated that aflibercept might improve the drainage function of RMG and help them flush the fluid out of the cell body, thus decreasing the intracellular edema of RMG.

**Table 5:** En-face areas of cystoid edema in segmentation of DCP before and after IAI in patients with DME.

Follow-up	Mean ± SD (pixels)	*n*	95% CI	*p*
Baseline	95760.94 ± 12913.65	16	-	-
1st IAI	86307.06 ± 16719.92	16	2022.38, 20930.13	0.100
2nd IAI	87927.18 ± 10398.46	11	5759.85, 20831.79	0.003
3rd IAI	91020.40 ± 13764.53	10	−532.66, 22647.26	0.059
4th IAI	79397.17 ± 11293.08	6	8788.93, 33816.74	0.007

Data are number or mean ± SD. Statistically analysis was performed between baseline and each IAI. *p* < 0.05 was considered statistically significant. IAI, intravitreal aflibercept injection and CI, confidence interval.

### The Number of Hyperreflective Foci Is Significantly Decreased After IAI

**Figure 6:**
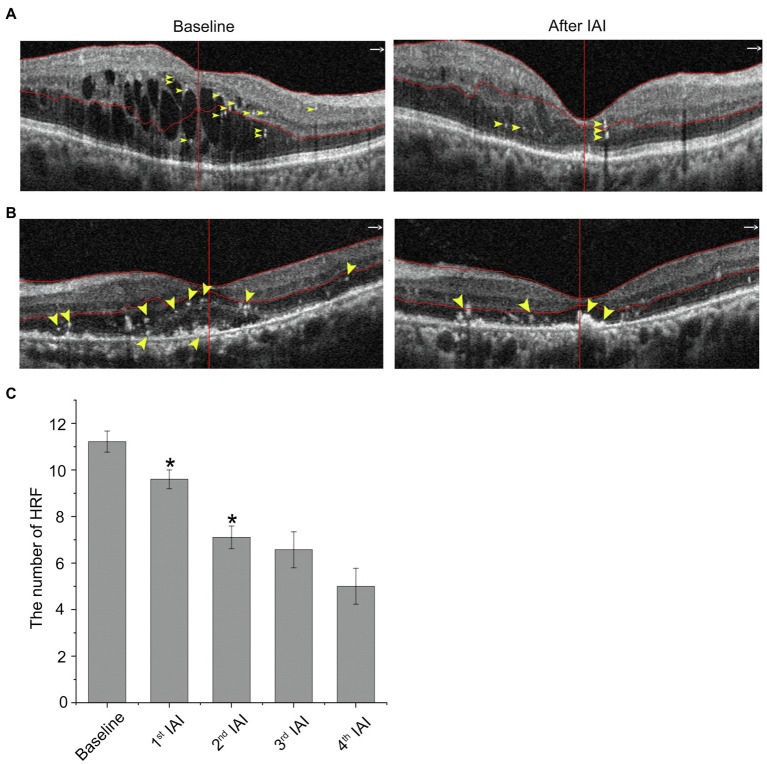
HRF are decreased after IAI. **(A)** Representative images of a 30-year-old male patient with DME at baseline and after a monthly IAI. Yellow arrowheads indicate HRF, mostly around the cysts. **(B)** Representative images of a 70-year-old male patient with DME at baseline and after a monthly IAI. Yellow arrowheads indicate HRF, mostly around the SRF. **(C)** Decreased number of HRF following IAI. Data are mean ± SD. ^*^p < 0.05 vs. baseline. HRF, hyperreflective foci; SRF, subretinal fluid; and IAI, intravitreal aflibercept injection.

Recently, HRF on OCT or OCTA have been considered active microglia/macrophages. In patients with DME, HRF were distributed across all retinal layers, including the inner and outer retina, and mainly located at the margin of cystoid edema (Figure 6A) or around the SRF (Figure 6B) in treatment-naïve patients. The number of HRF was decreased after IAI both in the neural retina and SRF. Compared with baseline (11.22 ± 10.63, *n* = 23), the number of HRF was decreased after anti-VEGF treatments to 8.87 ± 9.38 (1st IAI, *n* = 23, *p* = 0.008), 6.53 ± 8.59 (2nd IAI, *n* = 17, *p* = 0.001), 6.57 ± 5.86 (3rd IAI, *n* = 7, *p* = 0.138), and 5.00 ± 3.56 (4th IAI, *n* = 4, *p* = 0.132; Figure 6; Table 6). The decreased HRF indicated the anti-inflammatory effect of aflibercept on microglia/macrophages in DME.

**Table 6:** HRF before and after IAI in patients with DME.

Follow-up	Mean ± SD	*n*	95% CI	*p*
Baseline	11.22 ± 10.63	23	-	-
1st IAI	8.87 ± 9.38	23	0.67, 4.03	0.008
2nd IAI	6.53 ± 8.59	17	2.31, 6.98	0.001
3rd IAI	6.57 ± 5.86	7	−1.84, 10.41	0.138
4th IAI	5.00 ± 3.56	4	−5.06, 23.56	0.132

Data are number or mean ± SD. Statistically analysis was performed between baseline and each IAI. *p* < 0.05 was considered statistically significant. HRF, hyperreflective foci; IAI, intravitreal aflibercept injection; and CI, confidence interval.

### Foveal Avascular Zone Size and Vessel Density in Deep Capillary Plexus Show No Significant Changes After IAI

To assess whether aflibercept affects the FAZ as well as VD, several parameters, including FAZ area and perimeter, and foveal VD in DCP, were analyzed. The results showed no significant changes in the above parameters after IAI vs. baseline values (Supplementary Tables S1–3), indicating the safety of intraocular aflibercept.

## Discussion

The pathogenesis of DME is complex and multifactorial, involving the breakdown of the BRB, drainage dysfunction of RMG and RPE, and inflammatory cells and factors (Daruich et al., 2018). Retinal hypoxia leads to increased hypoxia inducible factor-1 alpha (HIF-1α) and retinal cell dysfunction (Das et al., 2015; Usui-Ouchi et al., 2020). Increased fluid entry and impaired fluid drainage are both involved in DME (Coscas et al., 2013). Diabetes-associated retinal ischemia and hypoxia increase the expression of HIF-1α, which leads to increased expression of vascular endothelial growth factor (VEGF), a potent mediator of vascular permeability and neovascularization (Rattner et al., 2019; Li et al., 2020). The VEGF family includes VEGF-A, -B, -C, and -D, and PlGF in mammals, of which VEGF-A and PlGF are involved in BRB breakdown and inflammatory cell activation, leading to macular edema, inflammation, and retinal neovascularization, etc. VEGF can also activate VEGF receptors 1 and 2 (VEGFR1/2) on endothelial cells, upregulating intercellular adhesion molecule-1 (ICAM-1) and vascular cell adhesion molecule-1 (VCAM-1), enhancing leukocyte adhesion (leukostasis) and occluding retinal capillaries (Cunningham et al., 2019; Van Bergen et al., 2019), which causes transient non-perfusion and enhances endothelial cell damage in the retina. Besides, VEGF-A and PlGF activate microglia/macrophage *via* binding of VEGFR1/2 on these cells, inducing cell proliferation, migration and phagocytosis, as well as the release of inflammatory factors involved in the pathogenesis of DME (Liu et al., 2020).

Anti-VEGF therapy has revolutionized the treatment of DME and is effective in DME patients in terms of improved visual acuity (VA) and decreased macular edema. Currently, anti-VEGF treatment has become the standard of care and the first-line therapy in DME (Wells et al., 2015), and the patients benefit a lot from anti-VEGF drugs, including ranibizumab, aflibercept, and conbercept. As a fusion protein, aflibercept, specifically binding VEGF-A, VEGF-B, and PlGF, inhibits the downstream signaling of VEGFR1/2, thereby reducing BRB breakdown and angiogenesis and reducing macular edema and retinal neovascularization.

In this study, several inflammatory and microvascular parameters were evaluated by OCTA and compared between baseline and after IAI in patients with DME. The preliminary results showed that aflibercept treatment significantly improved visual acuity, decreased central macular thickness and cystoid edema, and reduced the height of subretinal fluid as well as the number of hyperreflective foci; meanwhile, no obvious effects on the area and perimeter of FAZ, and vessel density were detected. The decreased CMT and improved BCVA by IAI demonstrated that aflibercept effectively attenuates the leakage of retinal blood vessels, i.e., BRB breakdown, through binding of VEGF-A and PlGF.

Cystoid edema often reflects the drainage defect of RMG due to the misdistribution and reduction of ion and water channels on RMG caused by diabetes, while SRF accumulation usually indicates the dysfunction of RPE, including both outer BRB breakdown and the drainage defect of RPE due to channels and pump dysfunction. However, the present study was an observational study that the decreased cystoid edema and reduced SRF by IAI were indicative of improved drainage function of both RMG and RPE after aflibercept treatment. The functional measurements for the drainage function in RMG and RPE are suggested to be explored both *in vivo* and *in vitro* in further study.

Although the formation of cystoid edema remains debatable, the role of RMG in the formation of cystoid edema was evidenced by OCTA (Spaide, 2016). RMG, as specific macroglia in the retina, regulates ion and water homeostasis, mainly by inward rectifying potassium channels (Kir), especially Kir4.1 and AQP4 (Narayanan and Kuppermann, 2017), to flush fluid and ions out of the retinal parenchyma, maintaining the retina in a relative dehydrated state. RMGs are also the main source of VEGF apart from vascular endothelial cells (Stitt et al., 2016). Any retinal stress can induce drainage dysfunction in RMG and modify the expression and cellular distribution of Kir and AQP4 in RMG. In the diabetic retina, Kir4.1 distribution is altered in RMG, leading to Kir4.1 channel loss around vessels in DCP, since RMGs are the sole mediators of the macroglia–vascular interaction at DCP, where astrocytes are absent (Sun et al., 2020). This results in fluid accumulation in the inner nuclear layer (Spaide, 2016), contributing to cystoid edema. Besides, the abnormality of Kir and water channels, as well as osmotic swelling probably due to potassium accumulation within the cells, might also reflect intracellular edema in RMG (Chung et al., 2019).

The RPE, besides being a component of the outer BRB, plays a major role in the fluid homeostasis of the outer retina, pumping the SRF to the choroids by active transport. As a highly polarized epithelium, any retinal stress, e.g., diabetes, could alter not only the tight junctions, leading to outer BRB breakdown, but also the proper distribution of membrane transporters of RPE cells, causing drainage dysfunction, which jointly result in SRF accumulation. The reduced tight junctions (Xia and Rizzolo, 2017) and alterations in the expression pattern of aquaporins (Tran et al., 2017; Oosuka et al., 2020) in RPE were reported in diabetic retinopathy. In this study, the reduced cystoid edema (decreased en-face area on DCP segmentation) and clearance of SRF (decreased height of SRF) after treatment might indicate that aflibercept could also improve drainage function in RMG and RPE. In our recent work (Wang et al., 2021), we found that the increased VEGF-A as well as the decreased Kir4.1 and AQP4 expressions in diabetic retinas contributed to the intracellular edema of retinal Müller glia. Anti-VEGF treatment protected Müller cells from intracellular edema *via* upregulating the expressions of Kir4.1 and AQP4 through binding VEGF-A. It could also increase the protein expression of sodium-potassium-ATPase (Na^+^-K^+^-ATPase), thus decreasing the intracellular sodium level to reduce the osmotic pressure of Müller cells, consequently preventing intracellular edema. In addition, other ion channels and aquaporins might also be involved in ionic homeostasis and water regulation of RMG and RPE. Thus, it merits further study to explore the detailed mechanisms of anti-VEGF reagents on the drainage function of both RMG and RPE.

HRF in the retina detected by OCT or OCTA have been reported in many retinal diseases (Vujosevic et al., 2017; Romano et al., 2020; Waldstein et al., 2020). Although the origin of HRF remains controversial, activated microglial cells are among the suspected causes (Coscas et al., 2013; Nagasaka et al., 2018). HRF are considered a biomarker of retinal inflammation (Zur et al., 2018). Microglial cells are the major resident immune cells in the retina and become activated once homeostasis is disturbed in the retina. Chronic hyperglycemia and low-grade inflammation induce macrophage recruitment and microglia activation (Madeira et al., 2015; Vujosevic et al., 2016; Toto et al., 2017). Moreover, following activation, the cell bodies of microglia become larger and are therefore more likely to be detected as hyperreflective dots or foci on OCTA scans (Yu et al., 2015). In a recent study, we found HRF are decreased significantly after three consecutive anti-VEGF injections in patients with neovascular age-related macular degeneration (Wu et al., 2021). In the present study, a significant decrease in HRF number was also observed in patients with DME after IAI. Since HRF on OCT or OCTA scans are considered activated microglia and/or macrophages, the decreased HRF found in DME demonstrated that aflibercept also has an anti-inflammatory function to deactivate microglia and/or macrophages.

In this study, retinal vascular parameters, including the FAZ, FAZ perimeter, and VD, remained unchanged after intravitreal anti-VEGF injections, suggesting that anti-VEGF treatment is safe with no significant effect on existing retinal blood vessels, consistent with previous studies. However, the long-term effects of anti-VEGF on retinal blood vessels and neuroglia deserve further investigation.

The limitations of this study include a limited number of evaluated eyes, losses to follow-up, and short-term observation and follow-up. Therefore, large sample multi-center studies are needed to clarify the long-term efficacy of aflibercept in the treatment of DME. Another limitation is that OCTA has high requirements on the fixation ability of patients, and small alterations could affect the direct comparison of treatment groups. Besides, OCTA cannot detect an active leakage as observed by fundus fluorescein angiography (FFA). Thus, further studies combining OCTA with other means of examination, e.g., FFA, are required.

**Figure 7:**
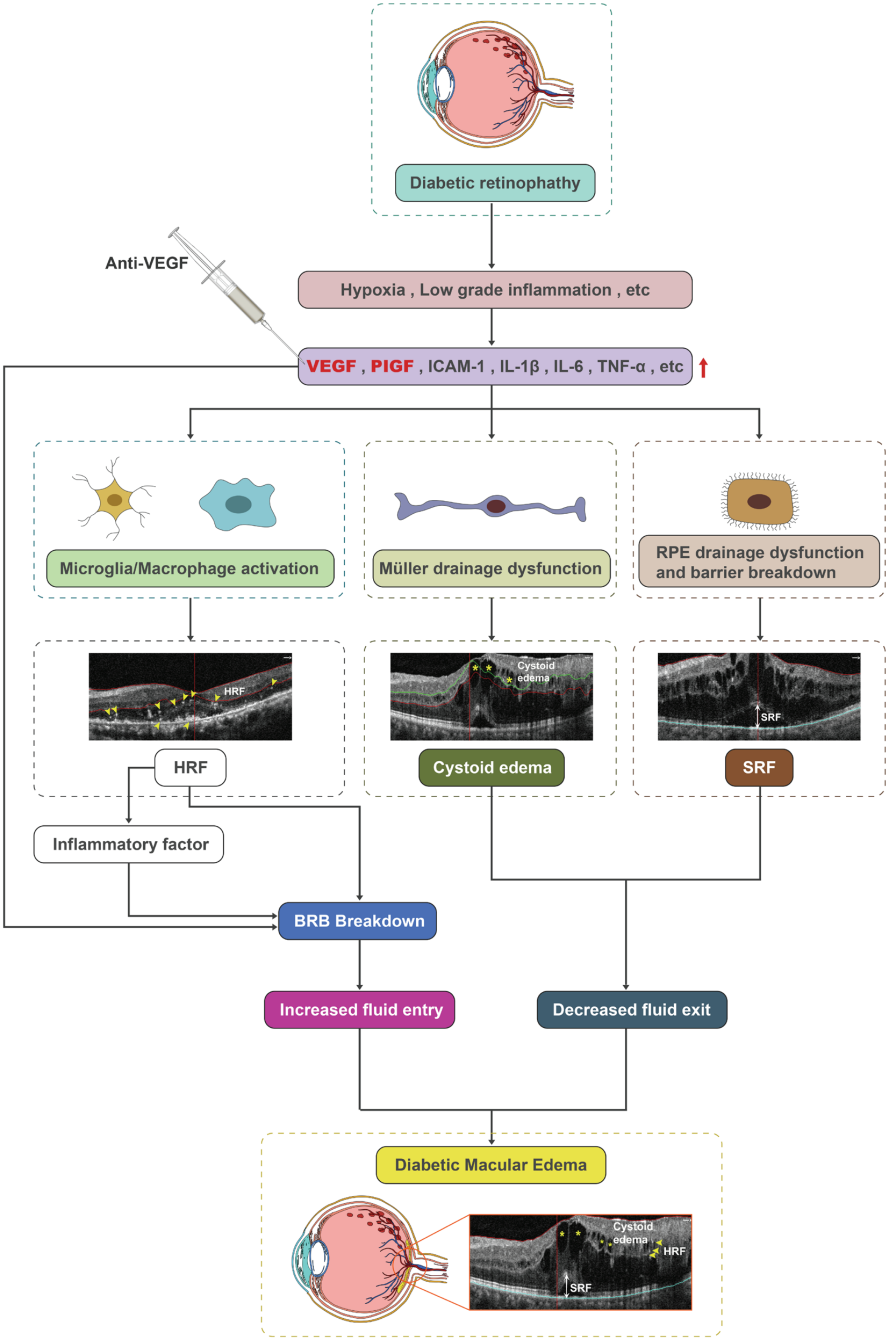
Schematic diagram depicting microglia/macrophage activation, the drainage dysfunction of RMG and RPE, and barrier breakdown as contributors to DME. Aflibercept could improve drainage function in RMG and RPE by decreasing cystoid edema and SRF, and repress inflammatory cells by decreasing HRF. DME, diabetic macular edema; HRF, hyperreflective foci; RMG, retinal Müller glia; RPE, retinal pigment epithelium; and SRF, subretinal fluid.

In conclusion, the pathogenesis of diabetic retinopathy and DME is very complex and remains to be fully elucidated. BRB breakdown, RMG and PRE drainage dysfunction, and inflammation are also involved in the pathogenesis of diabetic retinopathy and DME. Our preliminary data showed that besides its effect on the BRB, aflibercept could also improve drainage function in RMG and RPE by decreasing cystoid edema and SRF and suppress inflammatory cells by decreasing HRF. The protective effects of aflibercept occurred *via* binding of both VEGF-A and PlGF, thus repressing the downstream events of VEGFR1/2 in retinal cells (Figure 7). Yet, long-term observations with larger sample sizes and combined detection methods are required to confirm our findings.

## Data Availability Statement

The original contributions presented in the study are included in the article/Supplementary Material; further inquiries can be directed to the corresponding authors.

## Ethics Statement

This study was approved by the Clinical Research Ethical Committee of Shanghai General Hospital affiliated to Shanghai Jiao Tong University (Permit No. 2020KY205) and adhered to the principles of the Declaration of Helsinki. All individual participants provided written informed consent.

## Author Contributions

SQ, CZ, HQ, HX, DL, QQ, KL, and JingtZ contributed to the study concept and design, performance of the experiments, data review and analysis, and writing and editing of the manuscript. All authors contributed to the critical revision of the manuscript and gave their final approval of the submitted version. JingfZ and GX is the guarantor of this work and, as such, had full access to all the data in the study and takes responsibility for the integrity of the data and the accuracy of the data analysis.

## Funding

This research was supported by the National Natural Science Foundation of China (81870667, 81970810, 81970811, and 82171062), the National Major Scientific and Technological Special Project for “Significant New Drugs Development” during the Thirtieth 5-year Plan Period (2019ZX09301113), the Science and Technology Commission of Shanghai Municipality (19495800700), and the Domestic Science and Technology Cooperation Project of Shanghai Municipal Science and Technology Commission (21015800700).

## Conflict of Interest

The authors report no proprietary or commercial interest in any product mentioned or concept discussed in this article, as well as no conflict outside the current study.

## Publisher’s Note

All claims expressed in this article are solely those of the authors and do not necessarily represent those of their affiliated organizations, or those of the publisher, the editors and the reviewers. Any product that may be evaluated in this article, or claim that may be made by its manufacturer, is not guaranteed or endorsed by the publisher.

## Figures and Tables

**Table:** 

AQP4	Aquaporin 4
BCVA	Best corrected visual acuity
BRB	Blood–retinal barrier
CI	Confidence interval
CMT	Central macular thickness
DCP	Deep capillary plexus
DME	Diabetic macular edema
FAZ	Foveal avascular zone
FD	Foveal blood flow density
FFA	Fundus fluorescein angiography
HIF-1α	Hypoxia inducible factor-1 alpha
HRD	Hyperreflective dots
HRF	Hyperreflective foci
IAI	Intravitreal aflibercept injection
ICAM-1	Intercellular adhesion molecule-1
ILM	Internal limiting membrane
IOP	Intraocular pressure
IPL	Inner plexiform layer
Kir4.1	Inwardly rectifying K^+^ channel 4.1
logMAR	The logarithm of the minimum angle of resolution
OCTA	Optical coherence tomography angiography
PIGF	Placental growth factor
RMG	Retinal Müller glia
RPE	Retinal pigment epithelium
SCP	Superficial capillary plexus
SRF	Subretinal fluid
VCAM-1	Vascular cell adhesion molecule-1
VD	Vessel density
VEGF	Vascular endothelial growth factor
VEGFR	Vascular endothelial growth factor receptor

## Supplementary Material

The Supplementary Material for this article can be found online at: https://www.frontiersin.org/articles/10.3389/fphys. 2021.791442/full#supplementary-material
