# Supplementary material for: Hyperreflective Foci and Subretinal Fluid Are Potential Imaging Biomarkers to Evaluate Anti-VEGF Effect in Diabetic Macular Edema
Source: Front Physiol. 2021 Dec 23;12:791442. doi: 10.3389/fphys.2021.791442 (PMC8733589; doi:10.3389/fphys.2021.791442)
Supplement: Supplementary file 1 [file Data_Sheet_1.docx]

Supplementary Data：

Table S1. FAZ areas before and after IAI in patients with DME

| Follow-up | Mean±SD | n | 95% CI | P value |
| --- | --- | --- | --- | --- |
| Baseline | 0.28±0.12 | 11 | - | - |
| 1^st^ IAI | 0.3026±0.07 | 10 | -0.09, 0.05 | 0.503 |
| 2^nd^ IAI | 0.31±0.18 | 10 | -0.17, 0.09 | 0.516 |
| 3^rd^ IAI | 0.29±0.04 | 5 | -0.05, 0.10 | 0.451 |
| 4^th^ IAI | 0.23±0.13 | 3 | -0.16, 0.33 | 0.345 |
| 5^th^ IAI | 0.33±0.05 | 2 | -0.12, 0.04 | 0.185 |

Data are number or mean±standard deviation (Mean±SD). Statistically analysis was performed between baseline and each IAI. *P*<0.05 was considered statistically significant. FAZ : foveal avascular zone, IAI : intravitreal aflibercept injection, CI : confidence interval.

Table S2. FAZ-PERIM values before and after IAI in patients with DME

| Follow-up | Mean±SD | n | 95% CI | P value |
| --- | --- | --- | --- | --- |
| Baseline | 2.11±0.61 | 11 | - | - |
| 1^st^ IAI | 2.21±0.23 | 11 | -0.51, 0.31 | 0.602 |
| 2^nd^ IAI | 2.25±0.70 | 11 | -0.64, 0.43 | 0.661 |
| 3^rd^ IAI | 2.16±0.11 | 6 | -0.18, 0.61 | 0.215 |
| 4^th^ IAI | 1.83±0.57 | 4 | -0.75, 1.82 | 0.279 |
| 5^th^ IAI | 2.33±0.26 | 3 | -0.79, 0.61 | 0.623 |

Data are number or mean±standard deviation (Mean±SD). Statistically analysis was performed between baseline and each IAI. *P*<0.05 was considered statistically significant. FAZ: foveal avascular zone, IAI: intravitreal aflibercept injection, CI: confidence interval.

Table S3. Values of foveal VD in DCP before and after IAI in patients with DME

| Follow-up | Mean±SD | n | 95% CI | P value |
| --- | --- | --- | --- | --- |
| Baseline | 36.94±9.92 | 22 | - | - |
| 1^st^ IAI | 32.47±9.86 | 22 | -2.64, 11.57 | 0.205 |
| 2^nd^ IAI | 34.03±4.93 | 16 | -3.23, 8.79 | 0.34 |
| 3^rd^ IAI | 33.60±3.55 | 7 | -7.11, 5.74 | 0.803 |
| 4^th^ IAI | 29.65±5.35 | 4 | -14.51, 16.56 | 0.847 |
| 5^th^ IAI | 32.00±3.96 | 2 | -75.94, 67.64 | 0.597 |

Data are number or mean±standard deviation (Mean±SD). Statistically analysis was performed between baseline and each IAI. *P*<0.05 was considered statistically significant. VD: vessel density, DCP: deep retinal capillary plexus, IAI: intravitreal aflibercept injection, CI: confidence interval.
